# A new generation of health professionals – ethical dilemmas and challenges

**DOI:** 10.3325/cmj.2024.65.403

**Published:** 2024-10

**Authors:** Aleksandar Džakula, Dorja Vočanec, Karmen Lončarek

**Affiliations:** 1 Center for Health Systems, Policies and Diplomacy, Andrija Štampar School of Public Health, University of Zagreb School of Medicine, Zagreb, Croatia; *adzakula@snz.hr*; 2 University of Rijeka School of Medicine, Rijeka, Croatia

The beginning of autumn, as a time when new generations enroll in medical-oriented schools, is an opportunity for educators to reassess their materials and plans. It is also a good moment for them to evaluate the relevance of their messages to students. How well do their lectures keep pace with modern developments in the field, and how much positive energy can they convey to future health care workers? In this preparation, educators often face a dilemma as to which contemporary advancements in medicine should be highlighted. Additionally, they should decide on the best way to motivate new generations and strengthen their decision to pursue studies in health professions – professions in which they will, quite literally, be helping people.

While this task might seem noble, it brings increasing challenges each year, which educators, as motivators of new generations, must resolve. Should they direct the attention of the new generations to the patient or a particular diagnostic tool, disease, or therapy? Are we talking about groups of patients, the communities in which they live, or a broader ecosystem encompassing the healthy and the sick; professionals and laypersons; those who provide various services and those who finance them?

Concepts like patient-centered, person-centered, and people-centered care frequently appear, but it remains unclear how all these concepts can be reconciled and applied in practical situations to benefit both the patient and the health care system. The issue of available health care resources only increases the complexity. Common questions include: Is something public or private? Is there accountability for an action, and is profit possible (1)?

In addition to biomedical and public health challenges, modern health care systems are increasingly preoccupied with issues of investment development, return on investment, and profit generation through the provision of health care services. This business-oriented approach leads health care systems into the economic sphere, shifting emphasis away from human care to the care of capital. This shift is not necessarily negative in itself. However, when we direct and teach young people how to view the world, the emphasis on profit raises questions about the fundamental purpose of certain health care professions (2).

Although these questions about business and capital seem distinctly modern, their roots reach far back into history. The asymmetry of knowledge and power has always provided fertile ground for profitable business. In medicine, this is why we often find ourselves navigating a fine line between the needs of patients and the interests of service providers, suppliers, and vendors.

This dynamic is especially important when we try to direct young people toward health care professions. Future health care workers make a series of decisions throughout their professional development that not only determine what they will do in the future but also what values and principles will guide their careers. These decisions shape their relationships with their patients and colleagues, and the societal expectations that come with the responsibilities of health care professions. They influence whether health care will be approached with an emphasis on ethics, empathy, and the well-being of the individual, or whether direct economic parameters will take precedence (3).

A career in health care could be compared to a small mountain stream, which begins at its source, then flows down the mountain, gathering strength and eventually becoming a large, powerful river. The opportunity to influence this powerful river exists only at its beginnings, where small interventions can redirect its course, speed it up, or even change the side of the mountain it will flow down.

This metaphor of a mountain stream tells us that educators in medical schools and faculties have a special responsibility. They play a key role in presenting the possibilities and challenges related to health care professions to future health care workers. Perhaps it is precisely the small things we missed, or that we overemphasized, that will shape the professional and ethical behavior of future health care workers (4,5).

Thus, autumn in the cycle of educating future health care workers each year represents the spring of a new generation that will one day carry the mantle of future health care. As educators and professionals, we are not just passing on knowledge; we are shaping those who will one day be our colleagues and perhaps even those who will care for our health when we grow old (6).
